# Transfusion of fresh frozen plasma in non-bleeding ICU patients -TOPIC TRIAL: study protocol for a randomized controlled trial

**DOI:** 10.1186/1745-6215-12-266

**Published:** 2011-12-23

**Authors:** Marcella CA Müller, Evert de Jonge, M Sesmu Arbous, Angelique ME Spoelstra -de Man, Atilla Karakus, Margreeth B Vroom, Nicole P Juffermans

**Affiliations:** 1 Department of Intensive Care Medicine, Academic Medical Center, University of Amsterdam, Meibergdreef 9, 1105 AZ Amsterdam, The Netherlands; 2 Laboratory of Experimental Intensive Care and Anesthesiology (L.E.I.C.A.), Academic Medical Center, University of Amsterdam, Meibergdreef 9, 1105 AZ, The Netherlands; 3 Department of Intensive Care Medicine, Leiden University Medical Center, Albinusdreef 2, 2300 RC, Leiden, The Netherlands; 4 Department of Intensive Care Medicine, Tergooi Hospital, Van Riebeeckweg 212, 1213 XZ Hilversum, The Netherlands; 5 Department of Intensive Care Medicine, Diakonessenhuis, Bosboomstraat 1, 3582 KE, Utrecht, The Netherlands

**Keywords:** critically ill, transfusion, fresh frozen plasma, acute lung injury, bleeding, randomized clinical trial, TRALI, coagulopathy, INR, prevention

## Abstract

**Background:**

Fresh frozen plasma (FFP) is an effective therapy to correct for a deficiency of multiple coagulation factors during bleeding. In past years, use of FFP has increased, in particular in patients on the Intensive Care Unit (ICU), and has expanded to include prophylactic use in patients with a coagulopathy prior to undergoing an invasive procedure. Retrospective studies suggest that prophylactic use of FFP does not prevent bleeding, but carries the risk of transfusion-related morbidity. However, up to 50% of FFP is administered to non-bleeding ICU patients. With the aim to investigate whether prophylactic FFP transfusions to critically ill patients can be safely omitted, a multi-center randomized clinical trial is conducted in ICU patients with a coagulopathy undergoing an invasive procedure.

**Methods:**

A non-inferiority, prospective, multicenter randomized open-label, blinded end point evaluation (PROBE) trial. In the intervention group, a prophylactic transfusion of FFP prior to an invasive procedure is omitted compared to transfusion of a fixed dose of 12 ml/kg in the control group. Primary outcome measure is relevant bleeding. Secondary outcome measures are minor bleeding, correction of International Normalized Ratio, onset of acute lung injury, length of ventilation days and length of Intensive Care Unit stay.

**Discussion:**

The Transfusion of Fresh Frozen Plasma in non-bleeding ICU patients (TOPIC) trial is the first multi-center randomized controlled trial powered to investigate whether it is safe to withhold FFP transfusion to coagulopathic critically ill patients undergoing an invasive procedure.

**Trial Registration:**

Trial registration: Dutch Trial Register NTR2262 and ClinicalTrials.gov: NCT01143909

## Background

Fresh frozen plasma (FFP) is an effective therapy to correct for a deficiency of multiple coagulation factors [1]. International guidelines support its use in case of bleeding in patients with such a deficiency [2,3]. Use of FFP has grown steadily in the past years, in particular in the Intensive Care Unit (ICU) [4]. In the last decade, use of FFP has expanded to include prophylactic administration of FFP. However, there are concerns about the efficacy of FFP to prevent bleeding. Evidence from randomized controlled trials that support FFP transfusion to correct coagulopathy before an invasive procedure is limited, including commonly performed procedures on the ICU, such as insertion of a central venous catheter, a chest drain or a percutaneous tracheotomy [5]. Moreover, retrospective studies suggest that the risk of bleeding after an invasive procedure is low and relevant bleeding requiring blood transfusion or an intervention is less then 1% [6].

Prophylactic FFP may not further reduce bleeding incidence, but carries the risk of acute lung injury, occurring in up to 30% of transfused ICU patients [7], resulting in an increased duration of mechanical ventilation and ICU stay [8,9]. However, despite the absence of evidence, in our ICU patients, 33% of plasma is transfused to non-bleeding patients [10], which is in accordance with reports on ICU transfusion practice in Europe and the United States [11,12].

With the aim to investigate whether prophylactic FFP transfusions to critically ill patients can be safely omitted, a multi-center randomized clinical trial is conducted in ICU patients with a coagulopathy undergoing an invasive procedure.

## Methods/Design

### Objectives and design

The primary objective of the 'Transfusion of fresh frozen Plasma In non-bleeding Critically ill' trial ('TOPIC' trial) is to assess whether omitting prophylactic FFP transfusion is non-inferior to prophylactic FFP transfusion (current practice) to prevent relevant bleeding in ICU patients with a coagulopathy, who are undergoing an invasive procedure.

The preferred design to demonstrate the non-inferiority of no FFP transfusion prior to an invasive procedure against FFP transfusion to prevent bleeding would be a double blind, placebo-controlled clinical trial. However, manufacturing a completely matched placebo in full compliance with the current Good Manufacturing Practice standards was not considered possible for this non-commercial, academic study. Therefore, we have chosen for a multicentre prospective, randomized, open-label, blinded end point evaluation (PROBE) design.

The Institutional Review Board of the Academic Medical Center - University of Amsterdam, Amsterdam, The Netherlands, approved the trial. The TOPIC trial is conducted in accordance with the Declaration of Helsinki and was registered on March 26 2010 at http://www.trialregister.nl with trial identification number NTR2262 and on June 14 2010 at http://www.clinicaltrials.gov with trial identification number NCT01143909.

### Consort Diagram

**Figure 1:**
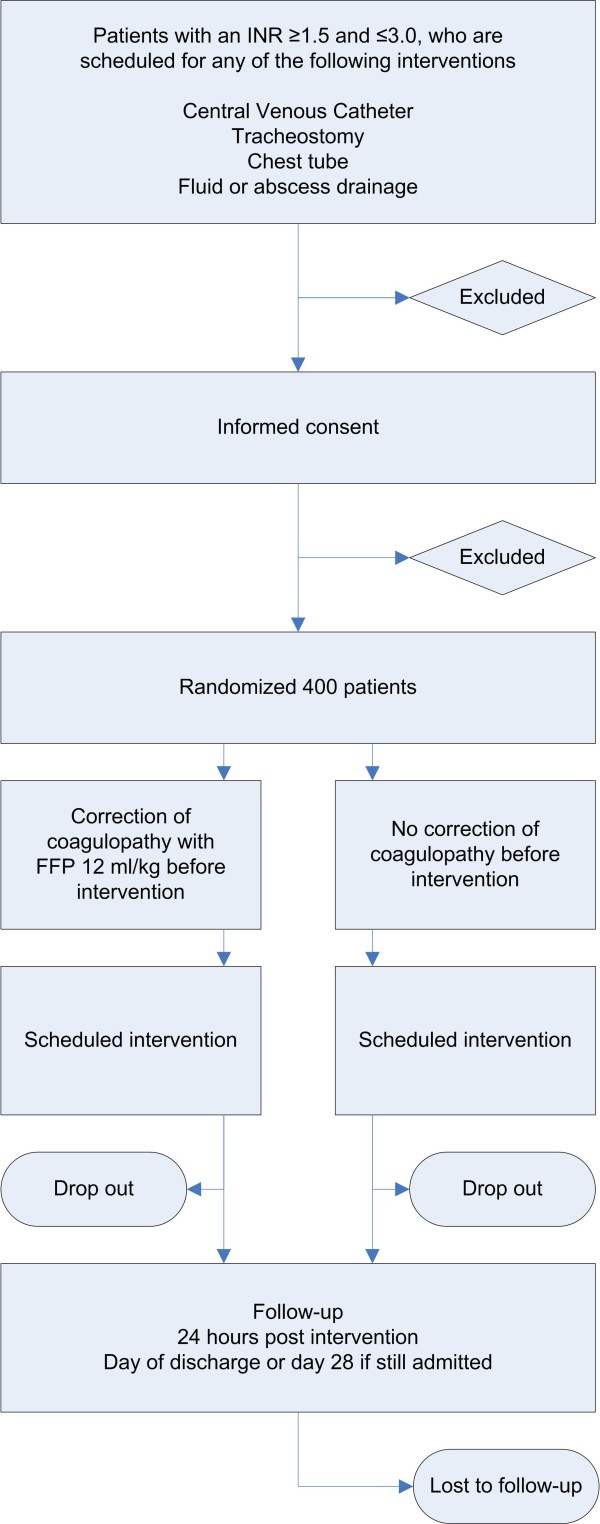
**CONSORT diagram**.

Figure 1 shows the CONSORT diagram of the TOPIC trial.

### Study population

The source population consists of patients admitted to medical-surgical ICUs, who have a prolonged International Normalized Ratio (INR) and will undergo an invasive procedure. Based on previous reports, we expect that 30% of ICU patients have a prolonged INR [13]. A total of 400 patients will be randomized to omitting FFP or receiving 12 ml/kg FFP before a scheduled intervention. Local investigators will screen consecutive patients with a prolonged INR, which is determined daily in all ICU patients. Patients with an INR ≥ 1.5 and ≤ 3.0, who are 18 years and older and will be undergoing an invasive procedure, are eligible. Defined invasive procedures are insertion of a central venous catheter, thoracocentesis, percutaneous tracheotomy or drainage of abscess or fluid collection.

Patients with clinically overt bleeding, e.g. with decrease of hemoglobin > 1 mmol/L, needing transfusion or hemodynamic instability due to bleeding at the time of the procedure or with a thrombocytopenia of < 30 × 10^9^/L are excluded from participation. Also, patients are excluded if they are treated with vitamin K antagonists, activated protein C, abciximab, tirofiban, ticlopidine, low molecular weight heparin (therapeutic dosage), heparin or prothrombin complex concentrates. In addition, patients with a history of congenital or acquired coagulation factor deficiency or bleeding diathesis are excluded. Patients will only be included after informed consent from the patient, his/her (legal) representative or the closest relative has been obtained.

### Randomization and intervention

Patients are randomly assigned to receive or not to receive a single dose of 12 ml/kg FFP. The randomization procedure is password protected, web-based, using permuted blocks and stratified by study centre and invasive procedure. Patients can only be randomized once (e.g. for one procedure). The units of FFP will be ordered from Sanquin, the Dutch National Bloodbank. The chosen amount of 12 ml/kg will lead to an estimated reduction of INR to 1.4 - 1.6.

### Standard procedures

The predetermined invasive procedures are placement of any central venous catheter, percutaneous tracheostomy, thoracocentesis and percutaneous drainage of any fluid collection or abscess. Clinical practices, such as using ultrasound guidance when inserting a central venous catheter, are applied according to each centers specific expertise and routine, to minimize interference of the trial intervention with normal clinical practice.

### Protocol drop out

Subjects can leave the study at any time for any reason if they wish to do so without any consequences. The investigator can decide to withdraw a subject from the study for urgent medical reasons. In patients who develop an acute reaction (increase in body temperature of more than 2°C, hypotension, bronchospasm or development of urticaria), the transfusion will be stopped immediately and adequate clinical measures will be taken accordingly With an incidence of transfusion reaction of 0.1- 1% [3], we expect little drop-outs. Unless patients declare that collected data need to be discarded, data of dropped out participants will be included in the intention to treat analysis.

### Rescue therapy

FFPs will be ordered (but not thawed) from the blood transfusion laboratory also for the patients randomized to the arm with no transfusion. When a relevant bleeding occurs during or after the procedure, these blood products will be readily at hand to be administered.

### Study end points

The primary outcome of this study will be a procedure-related relevant bleeding, occurring within 24 hours after the procedure. Relevant bleeding is defined using a validated tool (HEME) in the critically ill [14]: overt bleeding with any of the following: a decrease in hemoglobin by more then 2 g/dL (1.2 mmol/L) in the absence of another cause or transfusion of 2 or more units red cells without an increase in hemoglobin or a decrease in systolic blood pressure by more then 20 mmHg, or an increase in heart rate by 20 beats per minute or more, or bleeding at a insertion or wound site requiring an intervention. Interventions to cease bleeding are defined as an extra suture at the insertion site, embolization by an intervention radiologist or any surgical intervention required to stop the bleeding. The primary end point will be assessed by a physician who is blinded for the randomization result at 1 and 24 hours after the intervention.

Secondary endpoints include the occurrence of minor bleeding (e.g. prolonged bleeding at the insertion site or increase in size of subcutaneous hematoma), the development of ALI and circulatory overload. For this purpose the Lung Injury Score will be calculated at 24 hours. This score includes chest radiographic score, hypoxemia score (PaO2/FiO2), positive end expiratory pressure (PEEP) level (cm H_2_O), and respiratory system compliance score when available [15]. According to the definition, circulatory overload can be defined as bilateral edema on chest x-ray and a wedge pressure > 18 mmHg. In patients who will not have a SwanGanz catheter in place, circulatory overload will be defined as pulmonary edema in the presence of at least 3 of the following: positive fluid balance, elevated central venous pressure, a history of heart failure and increase in oxygenation in response to diuretics. Furthermore, the effect of FFP on correction of INR and clotting variables and the effect of FFP transfusion on outcome (duration of mechanical ventilation, length of ICU stay and ICU mortality) will be evaluated.

Other variables that are collected include demographics, co-morbidities, severity of illness (Sequential Organ Failure Assessment (SOFA) and Acute Physiology And Chronic Health Evaluation IV, APACHE IV [16]), medication use, hemodynamic and respiratory data (from the electronic patient data monitoring system). Blood samples will be drawn to determine coagulation variables, hemoglobin, blood gas analysis and liver- and renal function.

After the invasive procedure has been completed, repeated assessments will be made after 1 and 24 hours. These will include registration of hemodynamic and respiratory data from the Patient Data Management System (PDMS), a limited physical examination (inspection of the insertion site of the catheter or drain), inspection of drain production, and collection of a blood sample to perform blood gas analysis and assess hemoglobin levels. The limited physical examination will be done by an independent physician blinded for the randomization group. A chest radiograph will be performed 24 hours after the procedure.

### Statistical considerations

A systematic search of the literature showed that the occurrence of a relevant bleeding (resulting in shock or requiring blood transfusion or intervention) in patients with a coagulopathy undergoing invasive procedures is 0.1 - 0.8% [17-24]. Hence, it is expected that in 99% of the patients no major bleeding will occur under prophylactic FFP transfusion and that there will be no increase in the incidence of major bleedings in case no prophylactic FFP transfusion is given. Group size calculation is focused on demonstrating non-inferiority. When the sample size in each group is 198, a one-sided Z test with continuity correction (pooled) achieves 80% power to reject the null hypothesis that the proportion of bleeding patients in the experimental group (no FFP transfusion) is 0.04 or higher, i.e. is inferior to the proportion in the control group (FFP transfusion) by a safety margin of 0.03, in favor of the alternative hypothesis that the proportion in the experimental group is non-inferior. It is assumed that the expected difference in proportions is zero and the proportion in the control group is 0.01. The one-sided significance level of the test was targeted at 0.05. Based on our pilot trial, we expect that all included patients are available for evaluation at the end of the study. Therefore, we intend to enroll 200 patients per treatment arm: 400 patients in total.

### Statistical analysis

Baseline assessments and outcome parameters will be summarized using simple descriptive statistics. Variables will be expressed by their mean and standard deviation if normally distributed, if not normally distributed they will be expressed by their medians and interquartile ranges. The main analysis focuses on a comparison between the trial treatment groups of the primary outcome, the occurrence of relevant bleedings (using a binary variable), expressed in a relative risk estimate and absolute risk increase. In case of clinically relevant baseline imbalances, the primary outcome will be additionally analyzed using multivariate Poisson or logistic regression, when appropriate.

Secondary outcome parameters such as coagulation variables, acute lung injury, number of ventilation days and length of ICU stay will be expressed using continuous variables. Minor bleedings, occurrence of acute lung injury and serious adverse events will be expressed as binary variables (any versus none). The secondary outcome parameters will be analyzed using the Chi-square test (including RR estimates), two group t-test or Mann-Whitney test, when appropriate. In all analyses statistical uncertainties will be expressed in 95% confidence limits.

Because of the non-inferiority design of the trial, both an intention to treat analysis as well as a per-protocol analysis will be done.

### Study organization

The study principal investigator coordinates the study together with the local investigator of the Academic Medical Center. Each participating center has its own local principal investigator who all approved the final trial design and protocol.

This study is considered a low risk trial, in which current practice, transfusion of FFP, with known side effects, is compared to omitting FFP. However, an independent Data and Safety Monitoring Board (DSMB) is instituted and monitors patient safety during the study.

Any serious adverse event (SAE) will be reported to the Institutional Review Board of the Academic Medical Center - University of Amsterdam, Amsterdam, The Netherlands and the DSMB. A predefined list of SAEs will be listed and reported periodically instead of individually, including transfusion reactions and minor bleeding not requiring an intervention.

## Discussion

Coagulopathy is highly prevalent in critically ill patients. An INR of > 1.5 occurs in 30% of patients and is associated with increased mortality [13]. Risk factors for coagulopathy are sepsis, liver disease, multiple blood transfusions and trauma [13,25]. Coagulopathy results from an imbalance between activation of coagulation and impaired inhibition of coagulation and fibrinolysis. The disturbance between these components of the coagulation system leads to a variable clinical picture, ranging from patients with an increased bleeding tendency ("consumption coagulopathy") to those with diffuse intravascular coagulation (DIC) with (micro-) vascular thrombosis. However, clinical assessment of coagulation status and bleeding tendency in these patients is complex and the ability of global coagulation tests to accurately reflect *in vivo *coagulation is questioned [26]. In daily clinical practice, the INR, although initially developed to monitor warfarin therapy, is one of the commonly used tests to assess coagulopathy. In ICU patients, the majority indeed has a prolonged INR [10,11,13].

ICU patients frequently undergo invasive procedures, which carry the risk of bleeding. However, the incidence of bleeding after an invasive procedure in patients with a prolonged INR is low. It is shown that central venous catheter placement can be safely done in coagulopathic patients, without the need to correct coagulation abnormalities [19,21]. The incidence of major bleeding, requiring transfusion of red blood cells, ranges from 0.1-0.8%, of minor bleeding from 2.2-6% [20,22,27]. Studies on the safety of performing a percutaneous tracheostomy in patients with a prolonged INR report no evidence of major bleeding [28]. After thoracocentesis, the risk of relevant bleeding is 0.2% and the risk of minor bleeding is 2.6% [29].

Use of FFP to correct for a deficiency of multiple coagulation factors in case of bleeding [1] is supported by many international guidelines [3,30]. However, efficacy of the use of prophylactic FFP in non-bleeding patients is uncertain [31]. Although consensus regarding profylactic use is lacking, 30-50% of FFP units are administered to non bleeding patients [10,13,32]. There are important variations in ICU clinicians' beliefs and practices in relation to FFP treatment of non-bleeding coagulopathic critically ill patients [12,32,33]. Also, the dose of FFP administered varies widely, ranging from 7.2 to 14.4 ml/kg [32]. Studies on transfusion practice point to the assumption of ICU physicians that FFP corrects clinically relevant coagulopathy, thereby preventing bleeding [34].

FFP can correct a prolonged INR in critically ill patients, but most studies show a reduction and not a complete normalization of INR [1,7,35,36]. A minimally elevated INR (up to 1.5) cannot be corrected, even with higher doses of FFP [37,38]. When INR is > 1.5, a relationship between dose and target INR is found [1,38]. Studies on the dose of FFP to correct INR in ICU patients show conflicting results. Two randomized controlled trials (RCT) in ICU patients have been done. A small study showed that 33 ml/kg was more effective in achieving target levels of coagulation factors compared to 12 ml/kg [1]. However, the range of dosage in this study was wide and an association with bleeding was not studied. In a larger RCT in ICU patients, 20 ml/kg did not differ from 12 ml/kg in correction of INR or increasing coagulation factors above a minimum haemostatic threshold [35]. As complete correction of INR is not a target in this trial, because a mild prolonged INR (< 1.5) is not considered a risk of bleeding [6,37], we chose the dose of 12 ml/kg FFP for the control group. In addition, transfusion with higher doses of FFP is not current practice (own data and [12,32]) and may hamper compliance of physicians to the study protocol.

In recent years, many reports have been published on the detrimental effects of FFP. It is well shown that FFP transfusion is an independent risk factor for new onset of acute lung injury [39-45], in particular in patients who are mechanically ventilated [46]. Furthermore, studies consistently report a significant increase in length of ICU stay [34,44,46].

The TOPIC trial aims to demonstrate that omitting prophylactic FFP transfusion in coagulopathic critically ill patients undergoing an invasive procedure is safe in terms of bleeding complications. Recently, one other trial with similar purposes has been completed (http://www.clinicaltrials.gov NCT00953901), but results have not been published yet. Of note, in this trial, all hospitalized patients were eligible, in contrast to our trial which focuses on ICU patients. Recently, a trial on the effectiveness of FFP in critically ill has been completed (EPICC trial http://www.clinicaltrials.gov NCT00302965), not merely studying the effect of prophylactic FFP use since also active bleeding patients have been included in this study,.

A limitation of our trial is that it is not a double blind placebo controlled trial. However, manufacturing a completely matched placebo was considered not feasible for this non-commercial, academic study. The chosen PROBE design has the advantages of lower costs and greater similarity to standard clinical practice. Furthermore, there is no reason to believe that the reliability of end-point evaluation would differ between a PROBE study and a double-blind study, provided that the same criteria are applied [47]. In addition, it is shown that results from double-blind, placebo-controlled and PROBE trials are statistically comparable [48].

Former studies on the efficacy of FFP have been criticized by using irrelevant end-points, such as correction of INR [6]. The primary outcome of this study will be a procedure-related relevant bleeding, occurring within 24 hours after the procedure as assessed by the HEME tool [14]. A possible disadvantage of the tool is that some items scored with the HEME tool, such as decrease in systolic blood pressure or increase in heart rate, may also occur in the absence of bleeding. However, the inter-rater agreement and agreement between raters on bleeding severity assessments using the HEME tool was shown to be very high (φ = 0.99 (95% CI 0.97-0.99 and φ = 0.98 (95% CI 0.96-0.99 respectively) [14].

In conclusion, the TOPIC trial is a multi-center randomized trial, powered to test the hypothesis that omitting prophylactic FFP prior an intervention in critically ill patients with a coagulopathy is safe and is not associated with increased bleeding complications. In addition, the TOPIC trial determines the effect of FFP on correction of coagulopathy and evaluates the potential detrimental effects of FFP in this patient group.

## Trial status

The trial is currently ongoing; we expect to complete patient recruitment in 2013.

## List of abbreviations

ALI: Acute Lung Injury; APACHE IV: Acute Physiology And Chronic Health Evaluation IV score; CI: Confidence Interval; DSMB: Data Safety Monitoring Board; FFP: Fresh Frozen Plasma; HEME: HEmorrhage MEasurement; ICU: Intensive Care Unit; INR: International Normalized Ratio; PDMS: Patient Data Monitoring System; PROBE: Prospective Randomized Open-label Blinded-End point; RCT: Randomized Controlled Trial; RR: Relative Risk; SAE: Serious Adverse Event; SOFA: Sequential Organ Failure Assessment; TISS: Therapeutic Intervention Score System;

## Competing interests

The authors declare that they have no competing interests.

## Authors' contributions

MM and NJ: preparation of the initial drafts of the manuscript and preparation of the final version. All: review of the initial drafts of the manuscript. NJ and MM designed the study. All authors approved the final version of the manuscript.
